# First 3-D reconstruction of copulation in Lepidoptera: interaction of genitalia in *Tortrix viridana* (Tortricidae)

**DOI:** 10.1186/s12983-023-00500-4

**Published:** 2023-07-11

**Authors:** Boyan Zlatkov, Vladislav Vergilov, José Vicente Pérez Santa-Rita, Joaquín Baixeras

**Affiliations:** 1 Institute of Biodiversity and Ecosystem Research, Bulgarian Academy of Sciences, 1 Tsar Osvoboditel Blvd., 1000 Sofia, Bulgaria; 2 National Museum of Natural History, Bulgarian Academy of Sciences, 1 Tsar Osvoboditel Blvd., 1000 Sofia, Bulgaria; 3 Institut Cavanilles de Biodiversitat i Biologia Evolutiva, Universitat de València, Carrer Catedràtic José Beltran 2, 46980 Paterna, Spain

**Keywords:** Green oak leafroller, Genitalia, Functional morphology, Micro-CT, CLSM, SEM

## Abstract

**Background:**

The process of copulation in Lepidoptera is understudied and poorly understood from a functional perspective. The purpose of the present paper is to study the interaction of the male and female genitalia of *Tortrix viridana* Linnaeus, 1758 via three-dimensional models of pairs fixed during copulation. Other techniques (confocal laser scanning microscopy, scanning electron microscopy and histology) were used to clarify the role of the organs involved in the process.

**Results:**

Three-dimensional models based on micro-CT scanned copulating pairs were generated allowing visualisation of the position of the male and female counterparts, spatial changes during copulation, and the skeleto-muscular apparatus involved in the process. The male genitalia and their musculature are simplified in comparison with other lineages of the family, but the opposite is true for the female genitalia. The attachment of the couple is achieved only through flexion of the valvae, clasping the large and sclerotised sternite 7 of the female. The anal cone and socii of the male are in contact with certain parts of the anal papillae and the sterigma of the female. The long tubular vesica is inserted into the narrow posterior part of the ductus bursae. Its eversion is achieved by an increase in haemolymph pressure. A possible mechanism of stimulation of the female via pulsations of the diverticulum of the vesica was discovered. A compressed sclerotised area of the ductus bursae putatively serves as a valve controlling the transfer of ejaculated materials. Copulation progresses through two phases: in the first the vesica and its diverticulum are inflated by haemolymph, and in the second the diverticulum is not inflated, and the vesica is occupied by viscous ejaculated material. The formation of the multilayered spermatophore was observed, and we discovered that sperm is transferred very late in the copulation process.

**Conclusions:**

Copulation process in Lepidoptera is studied for the first time with three-dimensional reconstructions of couples of *Tortrix viridana*, used as a model species. The internal genitalia is the scenario of multiple interactions between male and female, but the external remain static. A possible mechanism of stimulation of the female internal copulation organs is proposed.

**Supplementary Information:**

The online version contains supplementary material available at 10.1186/s12983-023-00500-4.

## Background

The process of copulation in insects is highly variable among taxa and is understudied and poorly understood from a functional perspective. Even in an economically important order as Lepidoptera, details regarding the precise function of the organs and the structures involved in the process are known for only a small number of species [8]. Most Lepidoptera do not perform any special post-intromission courtship behaviour. Sexual interaction is thus concentrated in internal movements of difficult interpretation [13]. Various authors have investigated the process from different technical perspectives: interactions of macerated cuticular parts, whole fixed structures; and the skeleto-muscular complex [2, 4, 5, 14, 15, 20, 29, 30, 34, 39, 45].

Observing genital interaction during copulation is a difficult task. Researchers have applied various techniques, from the comparison of cuticular preparations or artificial pairing [7, 37, 48] to elaborate dissections and histological sections (above cited references). Perhaps the most accurate results can be achieved by the so called “serial morphology” proposed by Callahan [4] and based on dissection of subsequent stages. Instead of dissections, we generated three-dimensional (3-D) models based on copulating pairs fixed at different times during the process, which form the basis of the present study.

Initially applied for 3-D visualisation of the insect head [17], micro-CT scanning has been used in an increasing number of studies on insect morphology. The advantages of this technique for studying morphology are indisputable: the position of the organs is unchanged and 3-D models can be generated, enhancing the understanding of the function of the examined structures including in vivo approaches [46]. The first lepidopterous genitalia shown in detailed 3-D images were presented by Simonsen and Kitching [42]. However, the complexity of copulation in Lepidoptera has never been demonstrated with 3-D imaging based on pairs in copulation, though one could expect spectacular results.

The model insect of the present study is *Tortrix viridana* Linnaeus, 1758. It was selected for many reasons, particularly the average complexity of the genitalia. It can be easily collected in nature and reared ex situ. *Tortrix viridana*, commonly known as the green oak leafroller, is a major pest of oaks in the west-Palearctic region [3]. The host plant range of *T*. *viridana* is limited to the genus *Quercus* (Fagaceae). The species has a one-year life cycle, the imago flying in late spring and early summer. Larvae feed on tender shoots of oak in the initial stages and finish feeding upon the expanded foliage [11, 19]. Females oviposit on the bark of the host, where eggs hatch the following spring, completing the life cycle. Various details on the copulation in this species have been described [40], but the mechanism of the process from a morpho-functional aspect is completely unknown.

## Results

## Anatomy and interaction of genitalic structures

The anatomy of the cuticular parts is well known and described in numerous books in a taxonomic context. Here we emphasise the anatomy of those parts involved directly in the contact between the sexes. The structures are presented in dorso-ventral and postero-anterior arrangement.

### Male

**Fig. 1:**
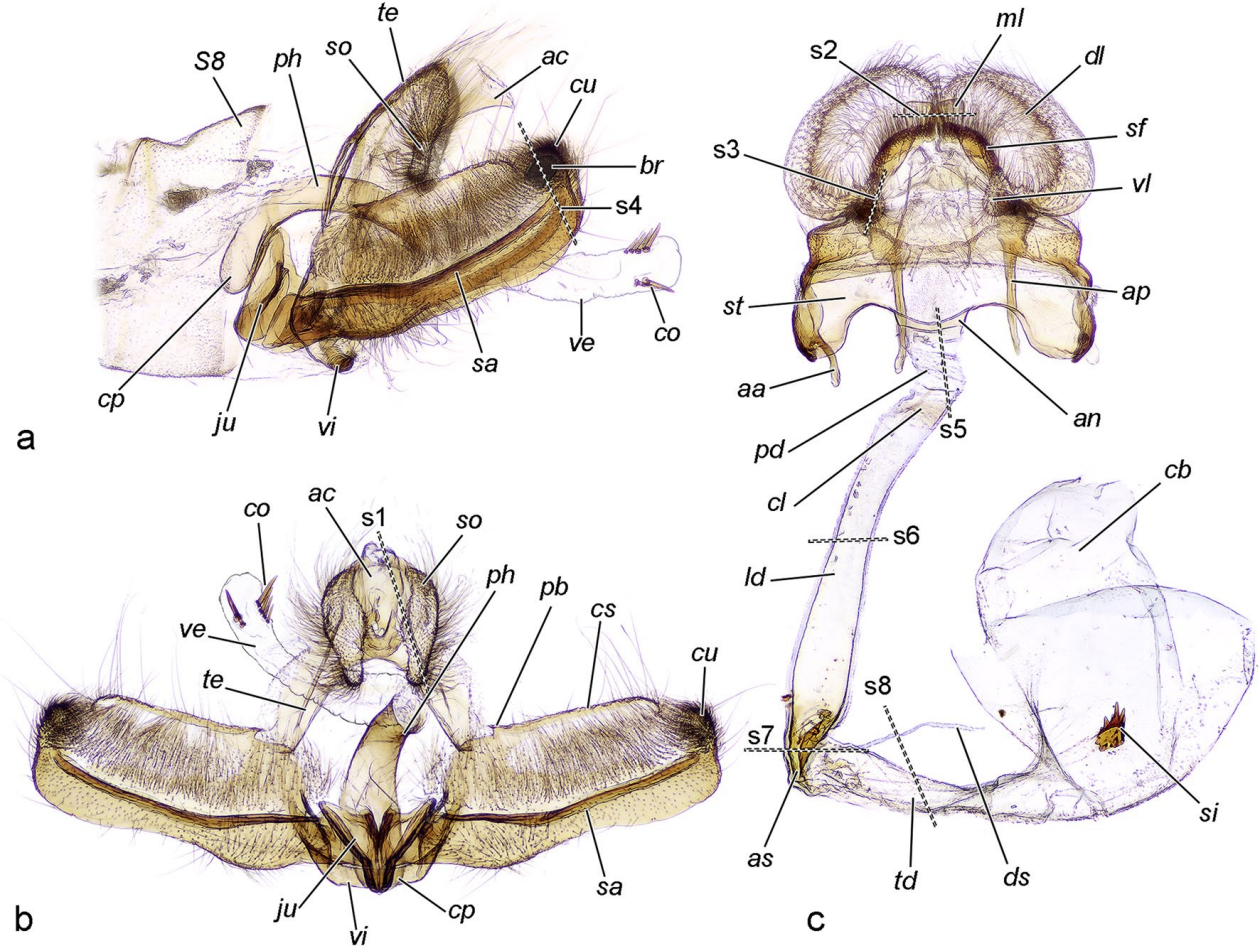
Cuticular skeleton of the genitalia (KOH maceration and staining (female only) with chlorazol black E), the darker the colour, the stronger the sclerotisation. **a** Male lateral (left) view with valvae flexed. **b** Male posterior ("open") view with valvae extended. **c** Female ventral view. s1–s8 planes of histological sections shown in Fig. 2

**Fig. 2:**
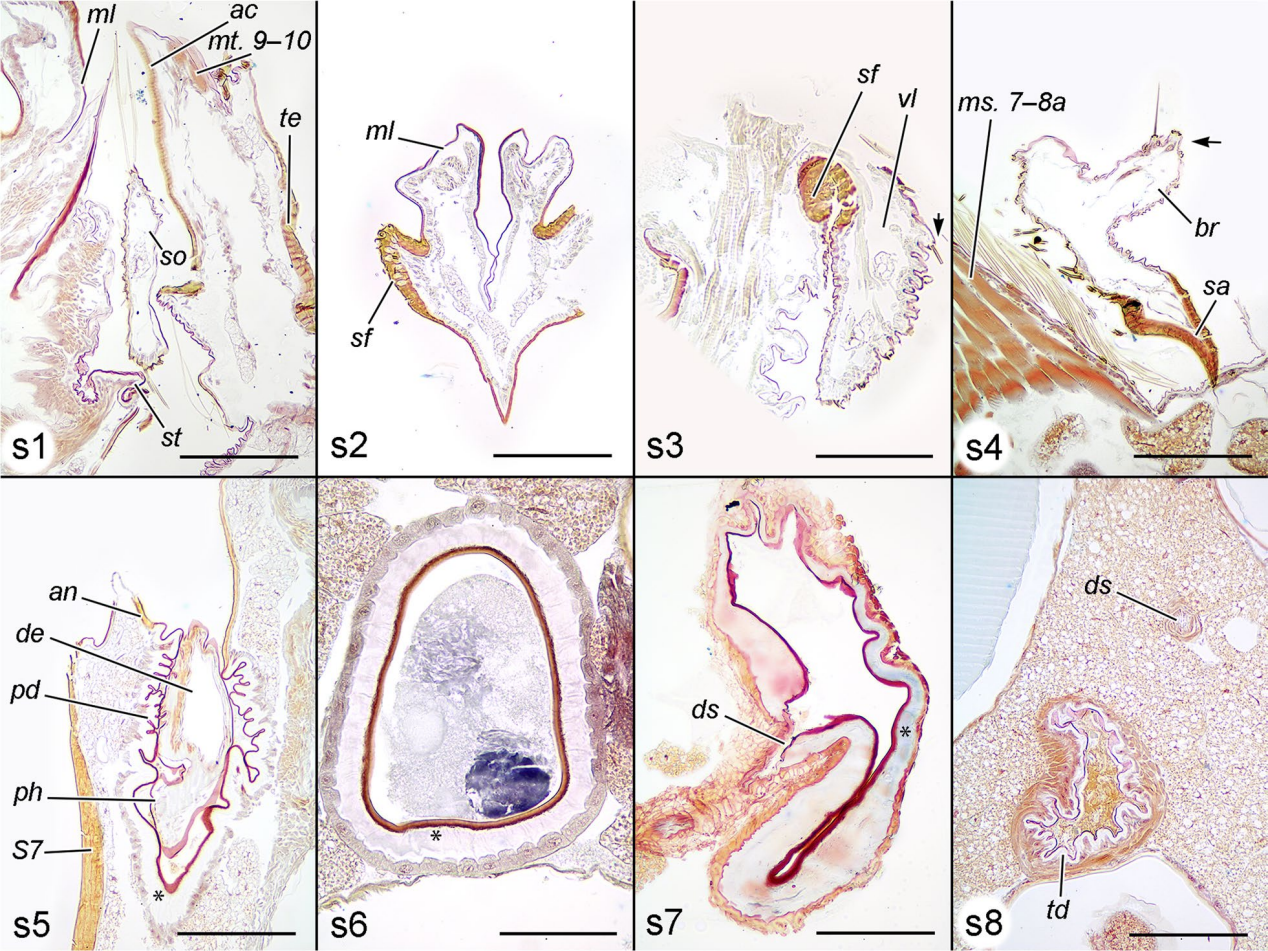
Histological sections of genitalia with details of certain structures. **s1**–**s8** planes of sections from Fig. 1. **s1** Parasagittal section through posterior ends of copulating pair (80 min after the onset of copulation). The anal cone of the male is approaching the median lobe of the anal papillae of the female, the socius is also in contact with the lobe and with the posterior margin of the sterigma. **s2** Nearly transverse (inclined to the frontal plane) section. **s3** Parasagittal section through ventral lobe of anal papilla. Thin folded cuticular membrane and numerous sockets of setae (probably sensilla trichodea) are visible. **s4** Parasagittal section through the distal part of valva in the area of brachiola of the male, and segment 7 of the female. Two sensilla trichodea are located at the distal part of brachiola, the sensory cell of the left sensillum is visible. **s5** Parasagittal section of posterior ductus bursae during copulation (20 min after the onset of copulation). **s6** Transversal section of longitudinal part of ductus bursae in the anterior area with thick endocuticle and exocuticle. Note the absence of muscles. Ejaculated material occupies the lumen (50 min after the onset of copulation). **s7** Transversal section of transitional area between longitudinal and transversal part of ductus bursae with ductus seminalis insertion of a virgin female. Note the presence of a muscular sheath. **s8** Parasagittal section of copulating pair 20 min after the onset of copulation. Ductus seminalis and transverse ductus bursae are cross-sectioned. Arrow (**s3**, **s4**) sensillum trichodeum, asterisk (**s5**–**s7**) thickened endocuticle. Scales: **s1**, **s2**, **s4**, **s5**, **s7**, **s8** 200 μm; **s3**, **s6** 100 μm

**Fig. 3:**
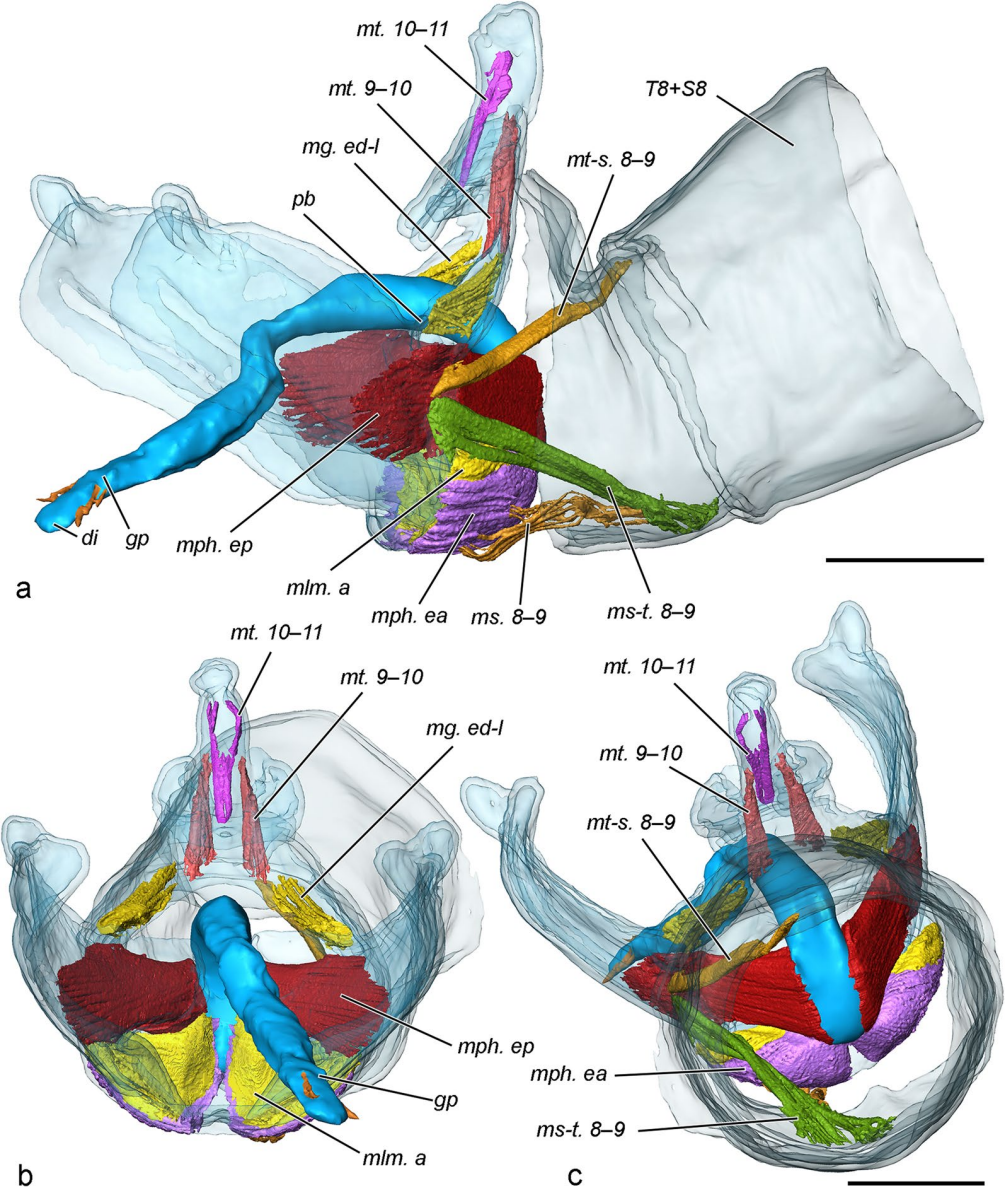
Musculature of male genital segments (pair at 20 min after the onset of copulation, volume rendering and false colour). **a** Lateral view. **b** Caudal view. **c** Frontal view. Scale bars 500 μm

(Figs. 1, 2, 3). Anal cone (= tuba analis auct.). The anal cone (*ac*) is a glabrous structure with vertical position, bowling-pin-shaped in postero-anterior view. It has a membranous dorso-apical extension and sclerotised lateral and posterior walls. During copulation it is in tight contact with the median process of the female’s papillae anales.

Socii. The socii (*so*) are large inverted drop-shaped pads consisting of membranous, densely setose cuticle. Numerous setae, sensilla trichodea and acanthae completely cover their surface. Their inner space is occupied mainly by fat body and haemolymph. The socii are in contact with the median and ventral lobes of the anal papillae, and also with the area between the anal papillae and sterigma, ventral sclerites of segment 8 and posterior margin of sterigma.

Valvae. In this species, the valvae are relatively simple, elongate, arched, almost rectangular plates. The lateral (“external”) surface is entirely covered by scales. The median surface is setose. The costal margin (costa, *cs*) is sclerotised, straight; the sacculus (*sa*) is large, strongly sclerotised, and its dorsal margin forms a longitudinal ridge. Distally and dorsally the sacculus forms a protuberance (sometimes referred to as cucullus, *cu*) densely covered by stout setae. The brachiola (*br*, a disto-lateral, thumb-like process) is also covered by scales and consists of a membranous cuticle with numerous setae and sensilla trichodea. It is filled with haemolymph and devoid of musculature. During copulation, the valvae grasp segment 7 of the female. Their flexion causes convex deformation of the *S7*, which is transferred to the sterigma, resulting in slight ventral displacement and exposing of the ostium. The cuculli of the flexed valvae are sunken within the invaginated pleurae of the female segment 7, ensuring stable suspension during the entire process. The function of the brachiola is unknown; during copulation it is pointed laterally.

Phallus. The phallus (*ph*) consists of a sclerotised tube and an internal membranous vesica (endophallus). The phallic tube is bent antero-ventrally at an angle of ca. 110° from its longitudinal axis and is anteriorly expanded into a rounded coecum penis. The vesica (*ve*) is entirely membranous, tubular when everted, with a slight widened basal part, almost twice as long as the phallic tube in maximal extension, apically with rounded, slightly flattened, pad-like diverticulum (*di*). The gonopore (*gp*) is located at the base of the diverticulum latero-dorsally on the right side. The diverticulum bears two rows of 3 (sometimes 2 or 4) modified setae: non-deciduous aciculate cornuti (*co*) pointed latero-apically and attached at right, at the side of the gonopore. The bases of the cornuti in each row are connected with a thick resilient exocuticle in a way that the cornuti forms two longitudinal combs. The vesica is everted into the tubular longitudinal part of female ductus bursae during the entire copulation. The cornuti can be inserted as far as the anterior sclerotised area of ductus bursae, reaching the flattened transitional part to the transversal part of ductus bursae.

Musculature (Fig. 3, Additional file 1). The male genitalic muscles of this species were described initially by Kuznetzov and Stekolnikov [25], but the anal cone is not present in their figure. The considerable reduction or complete loss of certain structures in *T. viridana* results in deviation from the ground plan.

*mt. 9–10* (musculus tergalis intersegmentalis 9–10) Depressors of uncus. In relation to a complete reduction of the uncus, the muscles are weak, diffuse bundles originating from the ventral arc of the tegumen and inserting into the anterior wall of the anal cone. Their flexion causes posteroventral displacement of the anal cone, pressing the papillae anales of the female.

*mt. 10–11* (musculus tergalis intersegmentalis 10–11) Retractor of the anal cone. *O* (origin): posterior basal wall of the anal cone; *I* (insertion): lateral apical walls of the anal cone. This small muscle was omitted by Kuznetsov and Stekolnikov (1973).

*mg. ed-l* (musculus gonopodialis externus dorsolateralis) Tergal extensors of valvae. *O*: dorsal area of lateral parts of the tegumen (*te*); *I*: *pb* (processus basalis valvae). These muscles are compact, thin bundles. Despite the name, their function in this species is flexion (Kuznetsov and Stekolnikov 1973) of the valvae (through the costa) contributing to the attachment with the female abdomen.

*mlm. a* (musculus laminae mediale anterior) Sternal extensors of valvae. *O*: juxta (*ju*); *I*: vinculum (*vi*). They are flat and largely covered by *mph. ea*. Their function is extension of the valvae.

*mph. ep* (musculus phallicus externus posterior) Protractors of phallus ("aedeagus" auct.). These are the largest genitalic muscles originating from the lateral walls of coecum penis (*cp*) and inserting into the basomedial area of the lateral wall of the valvae. They serve a double function: protraction of the phallus and flexion of the valvae, and have the main role on grasping the female. The considerable size of *mph. ep* is related to the complete loss of the primary medial flexors of valvae (musculus gonopodialis externus dorsomedialis, *mg. ed-m*) in *T. viridana* and a need to substitute their function.

*mph. ea* (musculus phallicus externus anterior) Retractors of phallus. *O*: ventrolateral walls of coecum penis; *I*: vinculum.

**Fig. 4:**
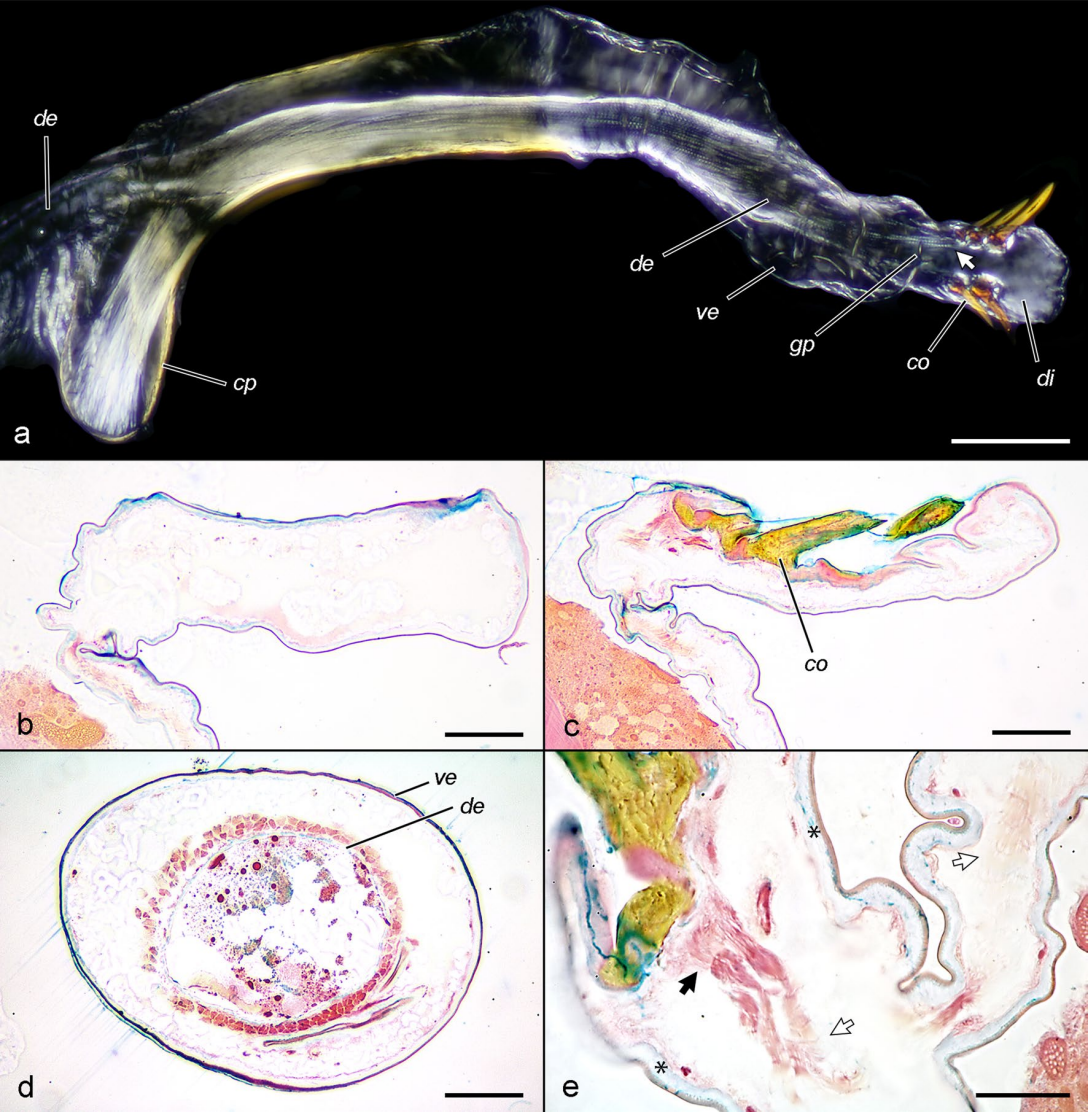
Structure of phallus of a male treated with DDVP. **a** Whole phallus in polarised light, *mph. il-t* glows strongly. **b** Longitudinal section of median area of diverticulum, note lack of any internal muscles. **c** Longitudinal section of diverticulum in the area of cornuti, the proximal two cornuti have muscle fibres attached, the exocuticle between sockets (red) is thickened. **d** Cross-section of the vesica, the intrinsic phallic muscle fibres form a sheath around the cuticular ductus ejaculatorius simplex (*de*). **e** Detail of **c** (rotated 90° anticlockwise in relation to **c**). white arrows muscle fibres, black arrow tendon cells. Scales: **a** 200 μm, **b**–**d** 50 μm, e 20 μm

*mph. il-t* (musculus phallicus internus longitudinalis) Intrinsic phallic musculature (Fig. 4). These consist of diffuse fibres originating from the coecum penis and inserting in the simplex, some of them in the distal parts of the vesica. A few fibres are attached to the base of the proximal cornuti, but not to other parts of the diverticulum.

The genitalia are retracted in the abdomen by the musculature of the preceding more generalised segment 8. The most prominent of them are:

*mt-s. 8–9* (musculus tergosternalis intersegmentalis 8–9) *O*: posterior margin of *T8*; *I*: articulation of the tegumen and vinculum.

*ms-t. 8–9* (musculus sternotergalis intersegmentalis 8–9) *O*: posterior margin of *S8*; *I*: ventrad the articulation of the tegumen and vinculum.

*ms. 8–9* (musculus sternalis intersegmentalis longitudinalis) *O*: *S8*; *I*: ventral area of vinculum, along with *mlm. a* and *mph. ea.*

### Female

**Fig. 5:**
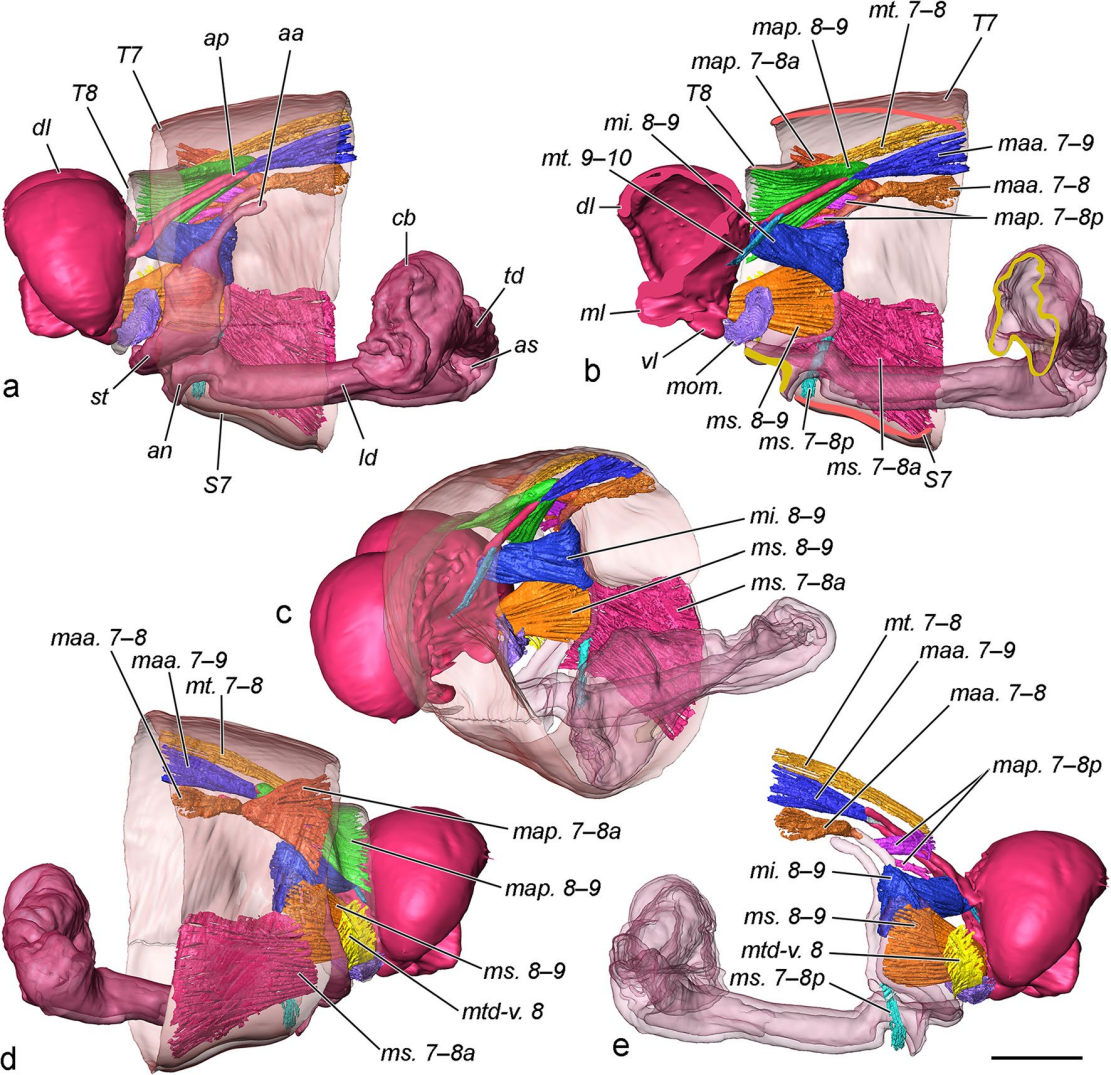
Musculature of female posterior abdominal segments (pair at 20 min after the onset of copulation, volume rendering and false colour). Only muscles of the left side shown, tergosternal and pleural muscles not presented. **a** Right view. **b** Right view sectioned. **c** Lateroposterior view. **d** Left view. **e** Left view with sclerites of segments 7 and 8 removed, *ms. 7–8a*, *map. 7–8a*, *map. 8–9* omitted. Scale bar 500 μm, all to scale

(Fig. 1, 2, 5). Papillae anales. The oviscapt (“ovipositor” auct.) in this species is floricomous (i.e., elaborated with long setae, some of them widened at the tip), with highly specialised anal papillae comprising several parts with different functions. They consist of two very large semicircular, dome-shaped dorsal lobes (*dl*) with numerous setae on the posteroventral surface, bordered ventrally by an arched, sclerotised fold (*sf*) bearing numerous stout setae; glabrous median lobe (*ml*) with gonopore and anus (dorsally to the gonopore) in the sagittal plane; and drop-shaped setose ventral lobe (*vl*). During copulation the median lobes of the papillae enclasp the anal cone and are in contact with the male’s socii. The ventral lobes interact with the socii as well. The dorsal lobes and sclerotised fold do not take part in interaction with the male but are displaced dorsally by the pressure of the anal cone of the male via the median lobes of the papillae.

Sterigma. The sterigma (*st*) is a ventral transverse sclerite, mostly covered with scales, with large rounded flat antero-lateral invaginations, an antero-median antrum (*an*) (narrow ring surrounding the ostium ductus bursae, *os*) and a setose posterior margin. Presumably, ventral elements of segment 8 and a portion of the apophyses anteriores are fused to form at least a part of the sterigma. The lateralmost parts of the structure protrude anterad into the apophyses anteriores (*aa*). The posterior setose margin interacts with the socii. During copulation the sterigma is deformed through compression of its lateral parts by the male’s valvae. This deformation causes opening of the ostium (tested with forceps on a freshly killed specimen).

**Fig. 6:**
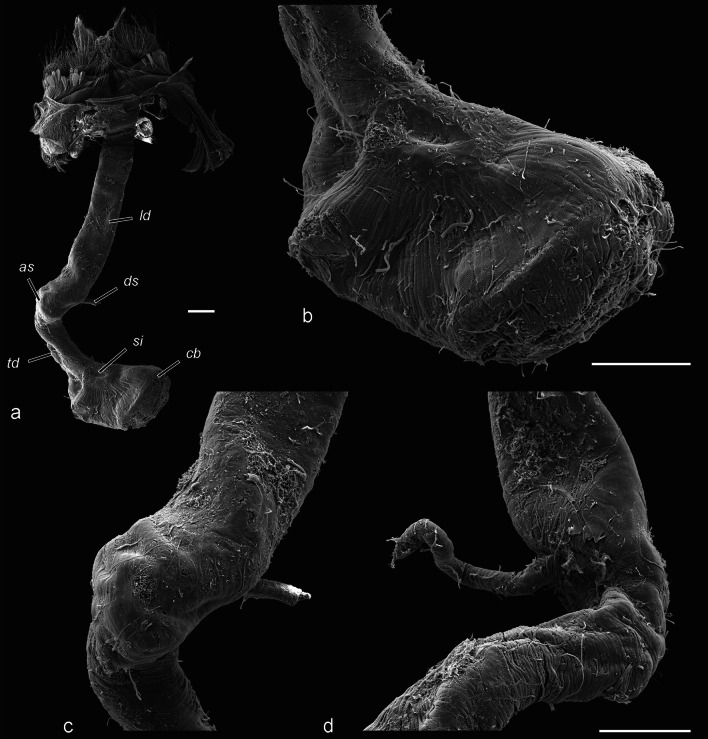
Bursa copulatrix (SEM). **a** Entire bursa copulatrix and sterigma. **b** Corpus bursae with muscle fibres irradiating from the signum. **c** Detail of anterior sclerotisation, lateral view. **d** Insertion area of ductus seminalis. Scale bar 200 μm, **c** and **d** to scale

**Fig. 7:**
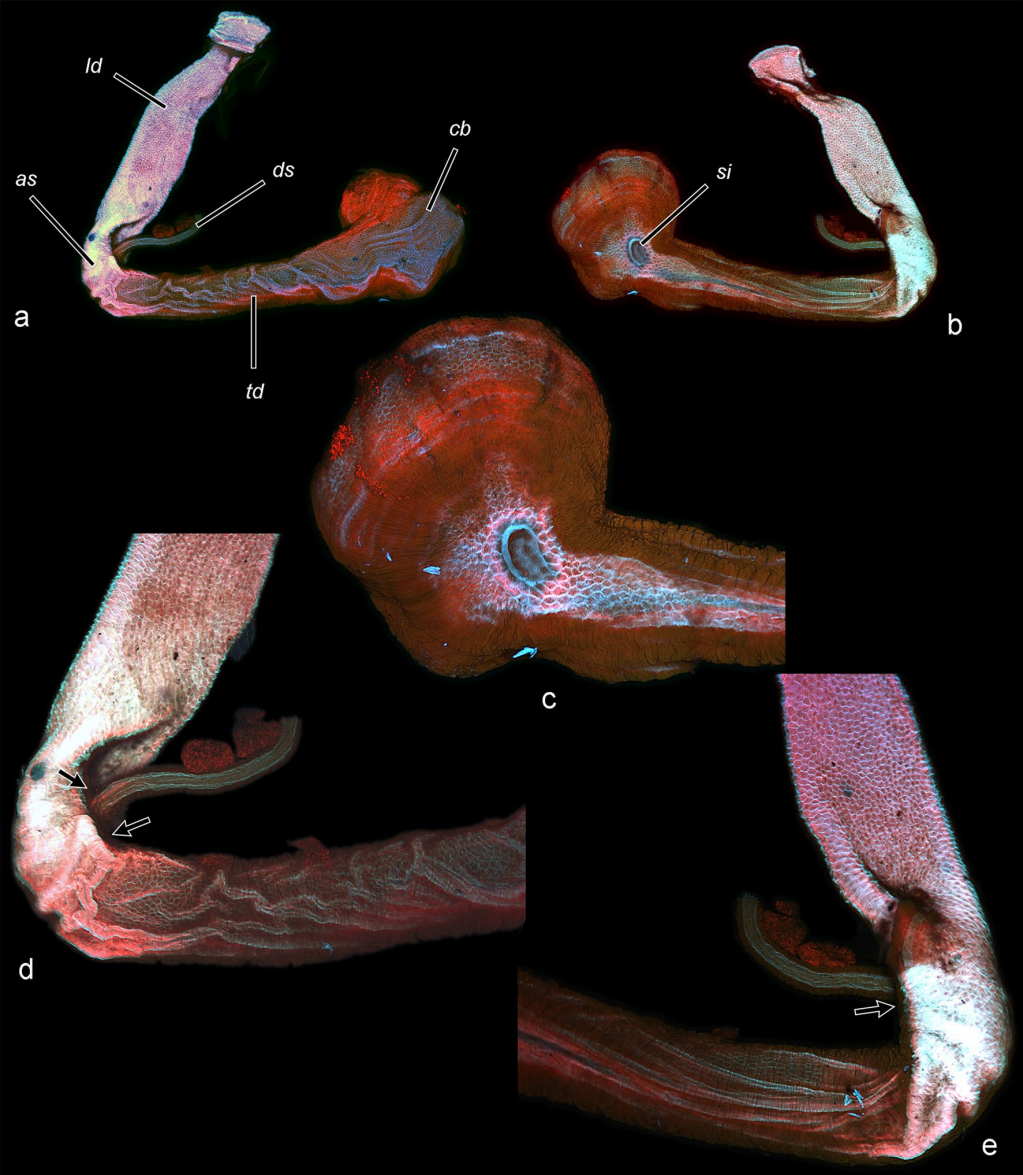
Musculature of bursa copulatrix (CLSM). **a** Ventral view. **b** Dorsal view. **c** Detail of corpus bursae with muscle fibres irradiating from the signum area. **d** Detail of ductus bursae, ventral view. **e** Dorsal view of **d**. Note the muscularis around the insertion area of ductus seminalis (arrows). Muscles in red, tendinous areas in blue, and the white colour corresponds with colour overlay of the fluorescent dyes and autofluorescence of the cuticle in all channels (red, green and blue)

Bursa copulatrix (Figs. 6, 7). This structure is divided into six sections. The posteriormost part is dorso-ventrally flattened, tightly sealing the genital tract in rest position. It consists of a relatively thin, transversely corrugated cuticle. The colliculum (*cl*) is a sclerotised complete ring and is manifested as a posterior sclerotisation of the next part. The longest component is the tubular longitudinal part of ductus bursae (*ld*) lined with thick endocuticle and moderately sclerotised exocuticle. This part of the bursa is slightly deviated diagonally to the left and devoid of muscles. Its function is to accommodate the phallus. Anteriorly the longitudinal ductus ends with the anterior sclerotisation (*as*), which is a corrugated, laterally flattened tube, more sclerotised than the previous part, with a concave right wall covered with muscle fibres. The diverticulum with cornuti is capable of reaching this part. After this area, the ductus bursae is sharply folded to the right, and in the inner curvature, the ductus seminalis is inserted. The anteriormost part is the transversal ductus bursae (*td*) with folded cuticle, slightly sclerotised near the anterior sclerotisation, otherwise membranous, with a thin muscularis consisting of annular fibres. The transverse ductus gradually widens into the corpus bursae (*cb*). No cervix is distinguished. The corpus bursae is drop-shaped, with folded (when empty) membranous cuticle and a signum (*si*) consisting of a heavily sclerotised plate with a bundle of spines (presumably large acanthae) located in the anteroventral part of the organ. The entire corpus bursae has a moderately developed muscular sheath, but in the area around the signum it is particularly thickened. The fibres radiate from the area around the signum.

Sternite 7 (*S7*). This sternite has a typical trapezoidal shape, has thick, strongly sclerotised exocuticle (unlike all other sternites), and is covered completely with scales. It changes from almost flat to semicylindrical by flexion of the valvae. Its deformation is transferred to the sterigma. The sternite plays a major role as a connecting structure during copulation.

Musculature (Fig. 5, Additional file 2). The musculature of the posterior abdominal segments of the female are mainly affected by the elaborate function of the oviscapt, and in *T. viridana* the latter is of a highly specialised floricomous type. Following is an outline of the muscles directly contributing to the movements of the oviscapt and sterigma. The pleural musculature is not studied in depth and not presented in the figures.

*mt. 7–8* (musculus tergalis intersegmentalis) Extends between the anterior margins of terga 7 and 8. It is a narrow muscle band with a role in retraction of segment 8.

*mt. 9–10* A very small muscle consisting of a few fibres. *O*: base of *ap*; *I*: rectal area of *ap*. This muscle is rarely observed in Lepidoptera, it is found in *Yponomeuta cagnagella* (Hübner, 1813) [24]. In this species, as well as in *T. viridana*, its point of origin is displaced to the base of the posterior apophyses.

*mtd-v. 8* (musculus tergalis intrasegmentalis dorsoventralis 8) *O*: posterior ventrolateral margin of sterigma; *I*: posterior ventrolateral margin of segment 8. A small, rectangular, thin muscle with oblique orientation. Its putative functions are retraction of the segment 8 and protraction of the sterigma. This muscle is designated as *ms. 8–9* in *Cydia pomonella* [24], but here the interpretation of Kristensen [23] is accepted.

*maa. 7–8* (musculus apophysalis anterior 7–8) *O*: anterolateral margin of tergite 7 (*T7)*; *I*: apex of *aa*. Relatively small muscle.

*maa. 7–9* (musculus apophysalis anterior 7–9) *O*: anterior dorsolateral margin of *T7*; *I*: apex of apophysis posterior. Relatively small muscle. Simultaneous contraction of *maa. 7–8* and *maa. 7–9* retracts the oviscapt.

*map. 7–8a* (musculus apophysalis posterior 7–8 anterior) *O*: posterior dorsolateral margin of *T7*; *I*: subapical area of *aa*. A large triangular muscle.

*map. 7–8p* (musculus apophysalis posterior 7–8 posterior) *O*: anterior dorsolateral margin of tergite 8 (*T8*); *I*: mediodorsal area of *aa*. This muscle consists of two bundles, here interpreted as branches of *map. 7–8* and both denoted as its posterior branch, *map. 7–8p*, but the homology is uncertain. Sometimes *map. 7–8* comprises two bundles, one of them located distinctly dorsad [23], and we speculate that this is the case in *T. viridana*, but the area of origin here is unusual and not previously reported: *T8*.

*map. 8–9* (musculus apophysalis posterior 8–9) *O*: posterior dorsolateral margin of *T8*; *I*: subapical area of *ap*. A large triangular muscle.

*mi. 8–9* (musculus interapophysalis 8–9) Extends between the basal apodemes of the unilateral apophyses. A large muscle with twisted bundles. The attachment on *aa* is in their middle (dorsoventral) part (i.e., the anterior margin of sterigma), some fibres turn around the margin and attach to the external wall of the sterigma. The latter three muscles (map. 7–8, *map. 8–9*, *mi. 8–9*) protract the oviscapt.

*ms. 7–8a* (musculus sternalis intersegmentalis longitudinalis 7–8 anterior) *O*: anterior margin of *S7*; *I*: anteroventral margin of sterigma. A large thin muscle covering most of the *S7*. Its function is retraction of the sterigma, and probably has an important role in juxtaposing the sterigma during copulation.

*ms. 7–8p* (musculus sternalis intersegmentalis longitudinalis 7–8 posterior) *O*: posterior medioventral area of *S7*; *I*: anterolateral angle of the sterigma. Very small muscle consisting of several thin fibres oriented transversely. Its putative function is abduction of *S7*, and may take part in copulation. The attachment points of the muscle suggest that it is a derivative of *ms. 7–8* and corresponds to the generalised scheme of Kuznetsov and Stekolnikov [24].

*ms. 8–9* *O*: medial anterolateral margin of sterigma on its internal wall; *I*: ventrolateral area of papilla analis. Relatively large muscle functioning as a ventral retractor of the anal papillae. The homology of this muscle follows Kristensen [23], it is denoted as *ma-s. 8–9* by Kuznetsov and Stekolnikov [24].

*mom*. (muscle of median oviduct) This delicate muscular mass is not a compact skeletal muscle but a part of the well developed muscular sheath of the distal median oviduct. It consists of fibres with various orientations and connects the oviduct wall with the ventral part of *T8*, and is probably related to oviposition.

## Stages of copulation

**Fig. 8:**
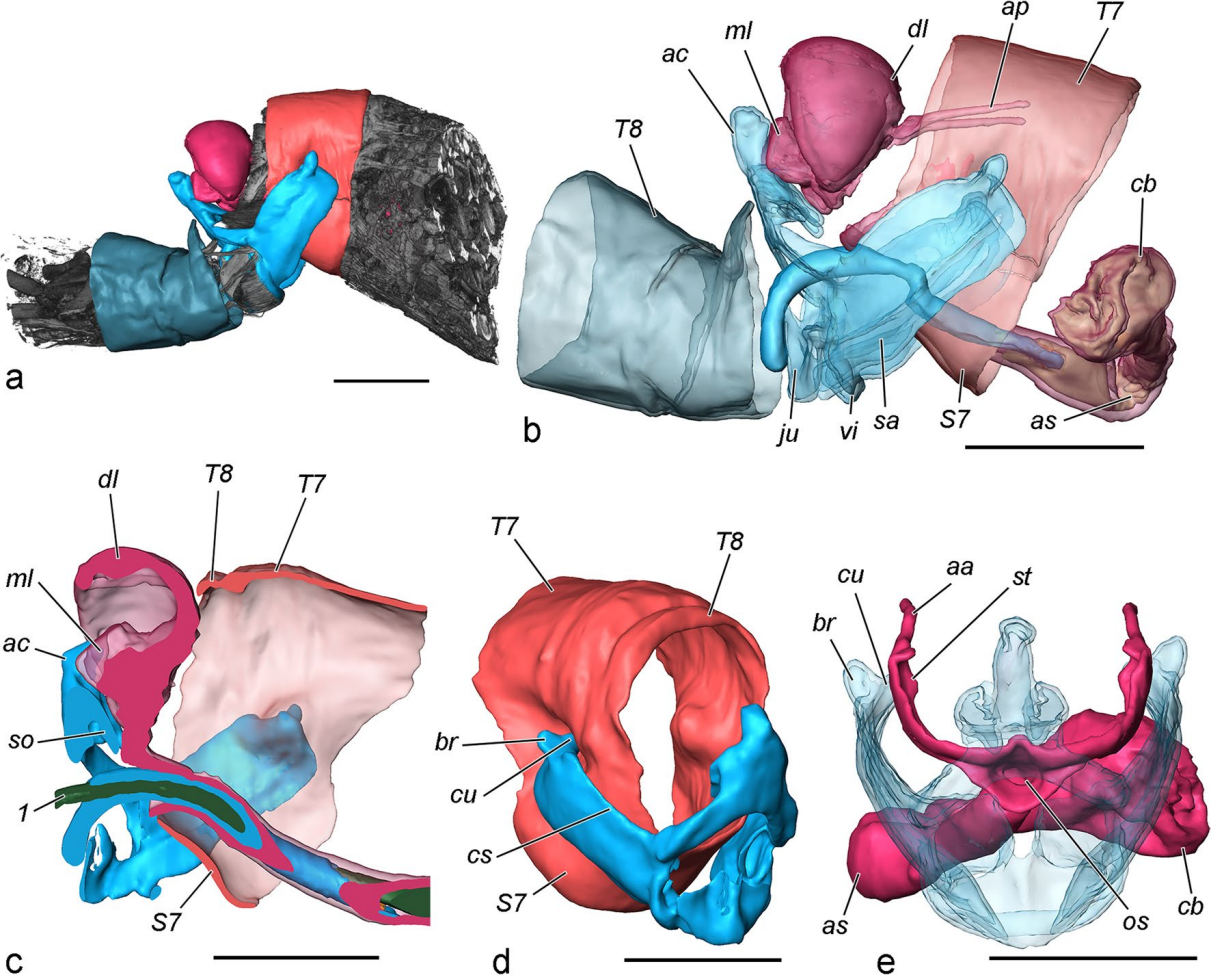
Alignment of genitalia during copulation (volume rendering of fixed couples, false colour). Male structures in blue tones, female structures in red tones. **a** Volume rendering of micro-CT scans of a whole sample (20 min after the onset of copulation) with reconstructed genitalia and some posterior abdominal segments. **b** Transparent view of genitalia (20 min). **c** Parasagittal section of a couple at 80 min after the onset of copulation, note the tight contact of the anal cone and socii with the median lobes of anal papillae and sterigma and relatively long distance between the juxta and *S7*. **d** Attachment during copulation. The cuculli of the valvae invaginate the pleurae of segment 7. **e** Deformation of sterigma caused by flexion of the valvae (20 min, postero-anterior view of the female). Scale bar 1 mm

**Fig. 9:**
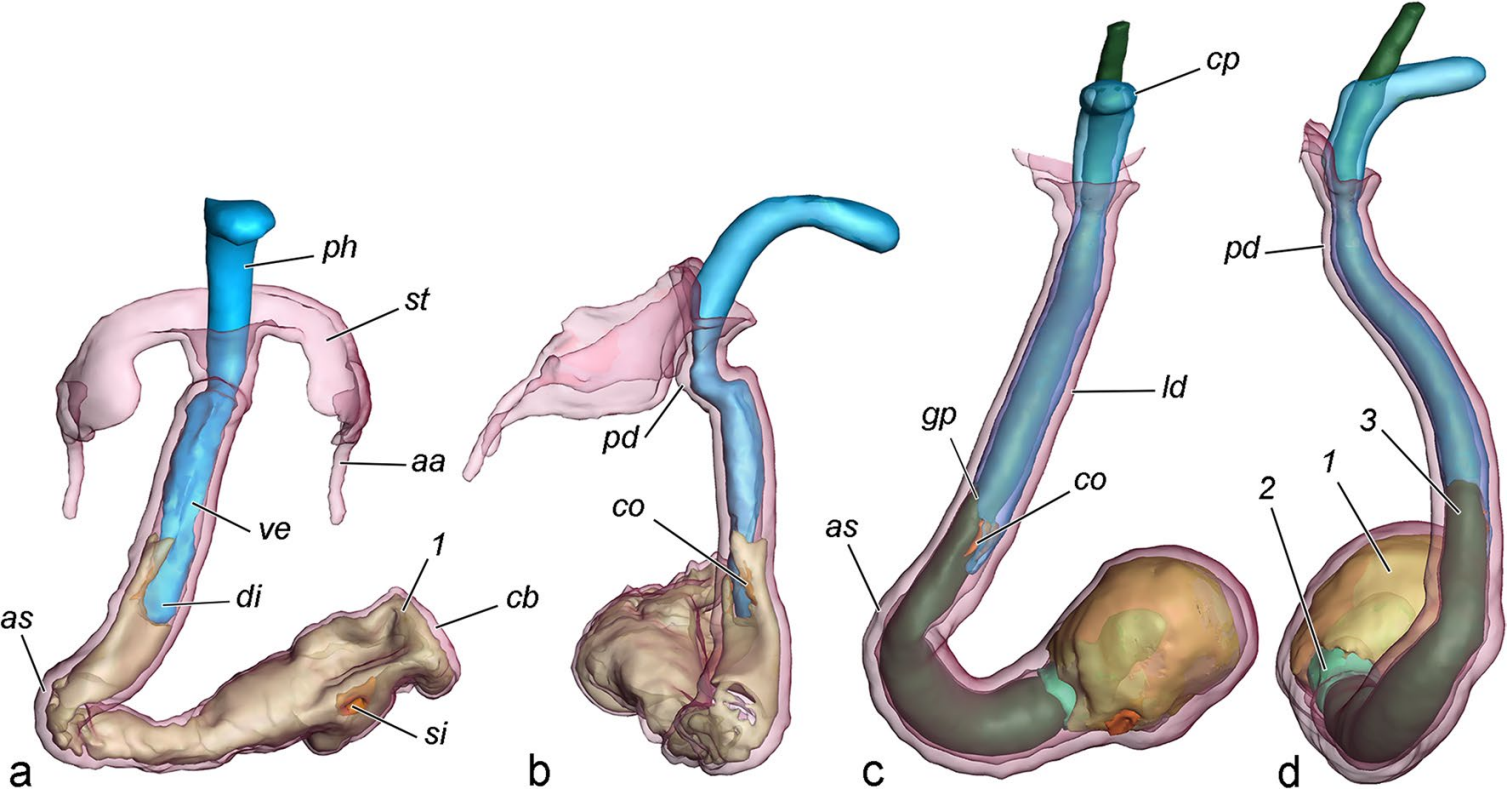
Two stages of internal genitalia during copulation (volume rendering and false colour). **a**, **b** Couple fixed at 20 min after the onset of copulation (first stage); the diverticulum and vesica are inflated by haemolymph, only semiliquid materials are transferred. **c**, **d** Couple fixed at 60 min after the onset of copulation (second stage; sterigma omitted); the diverticulum is relaxed; the rigid materials occupy the vesica and cause stretching of the curvatures of the ductus bursae. **a**, **c** Ventral view. **b**, **d** Lateral view. 1 corpus material, 2 sperm sack material, 3 collum material. Scale bar 1 mm

(Fig. 8, 9, Additional file 3)*.* Copulation starts with the juxtaposition of the genitalia of the pair and is a rapid process that is completed in a few seconds. The caudal ends of the animals align axially, and the valvae grip the posterior end of the female abdomen. No further details can be observed by external examination. The fixed pairs reveal a constant position of the external genitalia during the entire process, lasting about 90 min at 21 °C (two couples were left to complete the process). The anal cone compresses the median lobes of the anal papillae displacing them dorsally, and the socii are in contact with the ventral and median lobes of the anal papillae and the lateral parts of sterigma. The median concavity of the valvae corresponds to the convexity of *S7*, ensuring stable grasping and connection. The juxta was never observed in a tight contact with *S7*, but this may due to reaction to the fixative, and in reality, these structures are probably at least in close proximity to each other. Some of the couples demonstrated escape behaviour during the initial contact with the fixative (though only for a fraction of second), which caused observable separation of the genitalia, noticeable also in the internal genitalia.

The only changes during copulation are observed in the phallus and bursa copulatrix. The sclerotised phallic tube is probably inserted immediately after the onset of copulation, and then the vesica is everted. In the couples fixed at 10 and 20 min from onset, the vesica is everted completely into the longitudinal ductus bursae (although some retraction is visible in the 10th minute, which resulted from improper pouring of fixative and subsequent escape reaction); the posterior and transversal parts of the ductus bursae retain their folds; the vesica and its diverticulum are inflated with haemolymph; and the cornuti are pointed laterally, pushed into the anterior sclerotisation. The first ejaculated material (semi-liquid) is transported into the bursa during this time. In all subsequently fixed stages (30, 40, 50, 60, 70 and 80 min), the vesica is everted, but not inflated by haemolymph. Instead, its volume is occupied by the expanded cuticular simplex filled with denser, jelly-like ejaculate, particularly in the 80 min, when the collum (the most rigid of all materials) of the spermatophore is transferred. Hence, copulation can be divided into two stages: in the first, the vesica is inflated by haemolymph pressure, and the diverticulum also inflated; in the second, the vesica is occupied by ejaculate, and the diverticulum is relaxed. At the end of the second stage, the folds of the posterior and transversal ductus bursae are extended, and the anterior sclerotisation extends to a tubular shape with a rounded curvature. The volume of the corpus bursae increases considerably, and it gradually becomes subspherical.

The copulation process finishes suddenly by separation of the couple. No details of this stage are observed.

## Eversion in males treated with DDVP

**Fig. 10:**
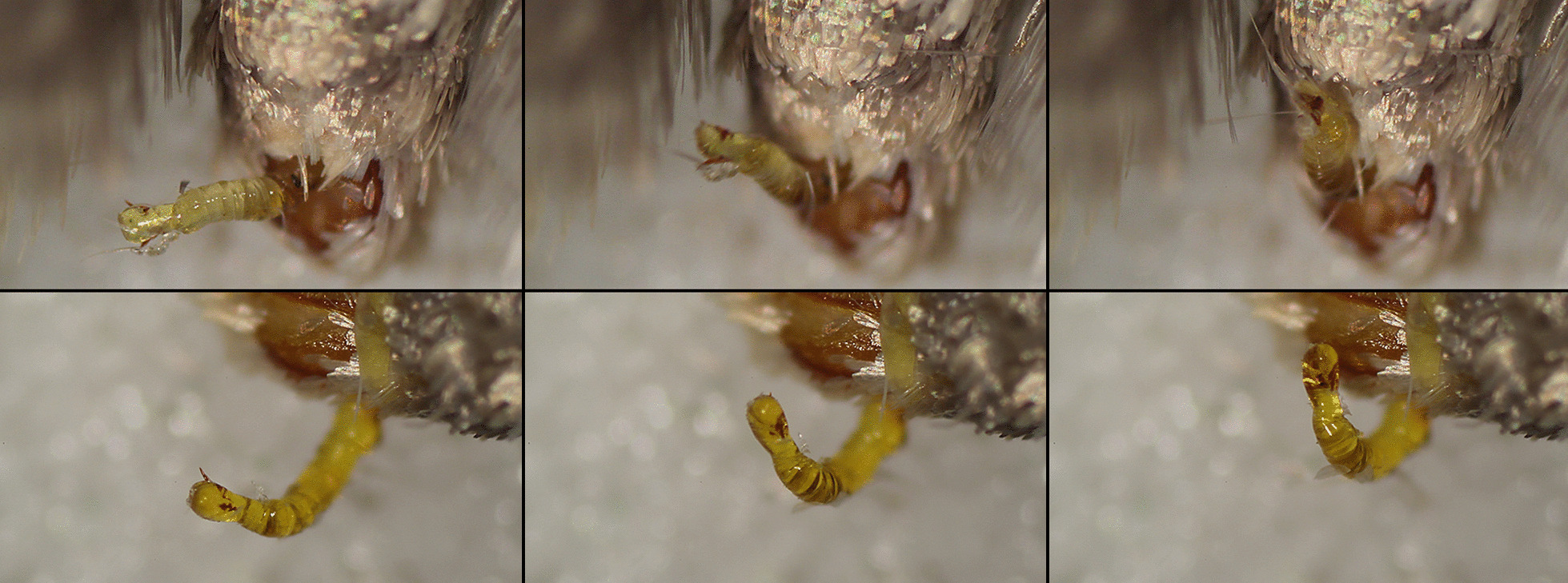
Movements of the vesica of two males treated with DDVP (video recording). Upper row ventral view, bottom row right view. Note the dorsal flexion of the vesica, inflated by yellowish haemolymph

(Fig. 10, Additional file 4). The eversion commences soon after the moth ceases erratic movements. The vesica looks transparent, inflated with a yellowish liquid. At the initial stage, only the basal part of the vesica is everted, then brown material which hardens quickly is ejaculated. The vesica everts slowly, at about 20 min from initiation, it is almost completely everted but not the diverticulum. Around 40 min from initiation, it performs repeating pulsating movements and bends dorso-laterally for about one second, then relaxes. The diverticulum inflates and pulsates as well at this stage, and the cornuti spread out at the moment of pulsation. After 60 min the hydrostatic pressure obviously decreases and the vesica is no longer inflated nor pulsating.

## Structure and formation of spermatophore

**Fig. 11:**
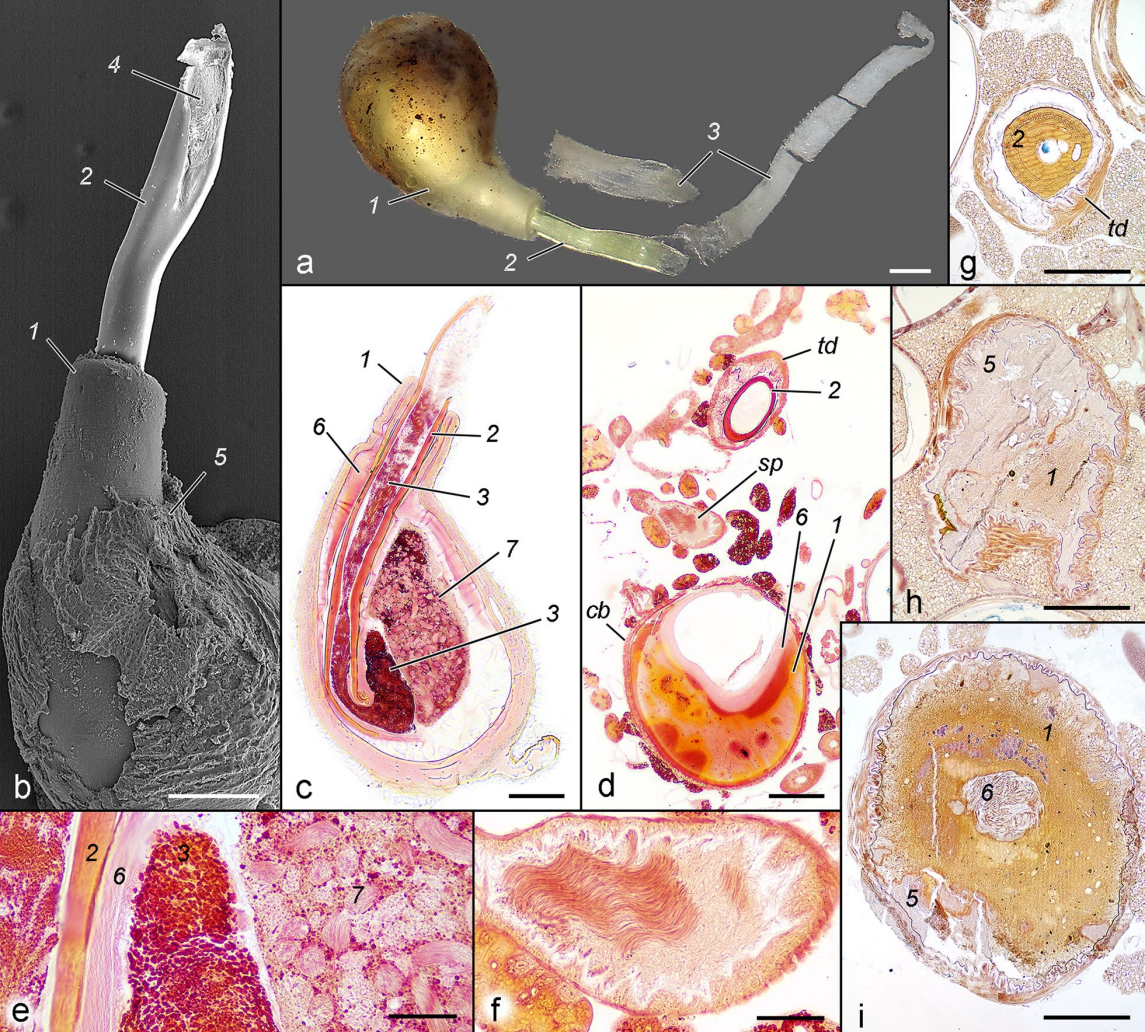
Structure of spermatophore. **a** Content of bursa copulatrix immediately after copulation (light microscopy). **b** Details of collum and corpus of spermatophore (the same sample from a, SEM). **c** Longitudinal section of spermatophore fixed immediately after copulation with spermatozoa in the sperm sack (brown material removed). **d** Partially digested spermatophore (sectioned tangentially) in the bursa of a female captured from nature. Spermatozoa have been transported to the spermatheca and the sperm sack looks empty. **e** Detail of c. **f** Detail of d showing spermatozoa in the spermatheca. **g** Couple fixed at 80 min, cross-section of the transversal part of the ductus bursae at the moment of transfer of the collum. **h** Couple fixed at the 20 min, parasagittal section in the area of signum; the brown material and corpus are not shaped yet. **i** Couple fixed at 60 min, parasagittal section in the area near the signum; the brown material has been displaced peripherally, the sperm sack is a compact fibrillar mass. 1 corpus, 2 collum, 3 white material, 4 aperture (obturated with white material), 5 brown material, 6 sperm sack, 7 sperm bundles. Scales: **a**–**d**, **g**–**i** 200 μm; **e**, **f** 50 μm

(Fig. 11)*.* Various materials are ejaculated into the female bursa copulatrix, some of which form a well-defined structure (spermatophore proper), while others appear as amorphous masses in the bursa and ductus bursae. Their transport determines the changes of all internal genitalic structures. The ejaculated materials were observed in males treated with DDVP, in fixed intact spermatophores and in sectioned samples, and in all cases, they show the same sequence of transportation. "Anterior" and "posterior" of the spermatophore here are used in relation to the corresponding parts of the bursa.

The completed spermatophore proper is a pear-shaped capsule with a thick collum sunk deeply into the corpus. The thick-walled corpus is occupied mainly by the sperm sack, containing sperm bundles. Six materials ejaculated during copulation can be distinguished, and they appear in the following order. The first (within 10 min from the onset of copulation) ejaculated portion is a relatively small volume of yellowish liquid containing dark brown granules of various sizes (here referred to as brown material). In samples fixed after copulation, the material forms a thin fragile crust enveloping the spermatophore corpus, and in the sections, it is stained pink. This material is followed (during 20–40 min from the onset of copulation) by the most voluminous material, which forms in the corpus. It is more viscous, and it is wedged into the brown material displacing it in the periphery of the bursa; it is stained orange to reddish in the sections and in the complete spermatophore is multi-layered. The third material (appearing 50–60 min after the onset of copulation) in the sections appears fibrillar to spongious, stained in pink, and forming the sperm sack. It is followed (during the 60–80 min) by the collum. The collum is the most rigid of all materials; during its transport it penetrates the sperm sack and drags the posterior end of the corpus, bending it around the point of penetration and forming the double wall of the posterior narrow part of the corpus. The aperture of the collum is wide, oblique as a syringe needle tip, facing the insertion of ductus seminalis. The sperm is transferred in the final stage of copulation (80–90 min) into the sperm sack (it was not observed in any copulating pair but only in a female fixed after copulation), increasing its volume. The last ejaculated material is a whitish liquid that occupies the collum, a part of the sperm sack, the entire ductus bursae (including the longitudinal region as far as the ostium), and embeds the free end of the collum. In the two fixed samples it appears opaque, fine-granular, easily disintegrating substance, and turns red after staining. With the progress of transferring materials, the corpus of the spermatophore gradually increases its volume and acquires its final shape. The materials are transported as compact masses, including the collum which has only a small lumen, but in the bursa they apparently experience some degree of condensation. In the completed spermatophore the materials of the corpus and sperm sack appear as hollow structures, and the collum as a thin-walled tube. The fate of the spermatophore was not studied in detail, but a female captured in nature demonstrates that the brown and the white materials have disappeared completely, the corpus has been partially digested, and the spermatozoa have been transported to the spermatheca (*sp*).

## Discussion

### Role of genitalia in the process of copulation

*Tortrix viridana* exhibits a typical “roof type” of copulation, i.e., the abdominal ends face each other with the heads of the insects pointing in opposite directions [31]. As in all known Lepidoptera, the male abdomen and female abdomen interact without external inversion. Counterintuitively, this results in the internal twisting of the internal genitalia and consequently they become asymmetric [8, 18].

Interactions between male and female during copulation are accomplished by both external and internal parts, but there is an important difference between these: while the internal ones experience certain changes, the external remain static. The function of the anal cone is to push the papillae anales in antero-dorsal direction via their median lobes, by contraction of *mt. 9–10* of the male; the opposite movement is accomplished by *mt. 10–11*. A possible reason is to protect these sensitive organs from the strong flexion of the valvae, though the female should be able to do this by her own musculature. The socii, being incapable of independent movement, simultaneously touch three structures of the female (median and ventral lobes of papillae and the setose margin of the sterigma), which may serve to provide information for the correct position of these parts, and/or to stimulate the moths. In fact, all these structures (apart of the median lobes) seem to be highly sensitive to mechanical stimuli, evident from the numerous sensilla trichodea on their integument. The functions of the valvae are presumably both mechanical (to maintain the female abdomen in a certain position, deformation of the sterigma to facilitate insertion of the phallus) and sensorial. The main role in grasping are performed by the sclerotised sacculi and particularly the cuculli, the latter gripping the lateral margins of the segment 7 via invagination of the pleurae. The flexion is achieved mainly by contraction of *mph.ep*, which are the largest muscles in the male genitalia, and probably to some extent by *mg. ed-l* [24]. The stout setae on the cuculli may serve to increase the friction with the female segment 7. The median surface of the valvae is covered by numerous setae, including sensilla trichodea, and probably provide information for the position of the female abdomen. The function of the brachiola is unequivocally tactile, but its role during the process is unclear. Being in the lateral subapical region and incapable of independent movements, it is not in contact with any other structure. It can be conjectured that the brachiolae may have a role in the initial stage of the copulation: the male attempting to locate the tip of the female abdomen may use them as sensitive "fingers". Once it finds the target, the male extends the valvae (probably via contraction of *mlm. a,* and possibly also *mph. ea* considering their spatial overlapping), approaches the tip of the abdomen, then grabs it and flexes the valvae. The flexion of the valvae not only grasps the female abdomen but also causes deformation of the sterigma, exposing the ostium. An interesting question is what the contribution of the female is during copulation in terms of movements of certain external structures. The sterigma is capable of at least some independent displacement via contraction of *ms. 8–9*, and especially *ms. 7–8*, which is particularly well developed and with two branches, in comparison with other Tortricidae lineages (e.g., *Cydia pomonella* (Linnaeus, 1758); [24], apparently in relation with the very large lateral parts of the sterigma. The highly specialised oviscapt needs elaborate and strong musculature, which is remarkably different from species with less specialised oviscapts. Additional branching was observed in the main protractor of the papillae anales, *map. 7–8*, which is particularly well developed in *T. viridana*, probably in relation with the large size of the papillae. These muscles may well take part in the copulation process as well, but this function is less evident from the three-dimensional reconstructions.

It should be noted that the male genitalic musculature in *T. viridana* is simplified, whereas the opposite is true for the female musculature, characterised by the development of additional branches with certain functions.

Unlike many species with well-developed dorsal clasping mechanism (e.g., uncus and gnathos or transtilla), *T. viridana* relies entirely on lateral clasping through the valvae for stable connection during copulation. The hypothesis for the connection function of the cornuti, at least in this species, should be discarded because of lack of any evidence. In another representative of Tortricidae, *Cydia pomonella*, the cornuti interact with similarly shaped spines, attached to a specialised region of ductus bursae (“cervix”) and attachment function was attributed to them [15].

The interaction between the external genital structures in *T. viridana* differs in many ways from other species [1, 10, 30, 35, 39, 41, 44], mainly due to considerable reduction of the dorsal male complex and development of large highly specialised papillae anales. Some similarities, e.g., holding segment 7 with the valvae as a result of reduction of the male dorsal complex, are observed in *C. pomonella* [15]. Interestingly, in *Cydia* Hübner the valvae have well sclerotised sacculi and relatively membranous costa but the papillae anales are not so bizarre. An independently developed floricomous ovipositor is also found in many Cnephasiini but male valvae have poorly sclerotised sacculi. Finally in the closely related genus *Acleris* Hübner male configuration is not so different but female ovipositors are discrete. All these configurations suggest a low level of correlation between the presence of a more or less developed ovipositor and the way the male grasps the female abdomen.

The function of the internal male genitalia can be split to two stages based on the viscosity of the ejaculated material. During the first stage, which is roughly one third of the duration of copulation, the vesica is inflated by haemolymph, and the diverticulum with its cornuti is engorged and reaches the anterior sclerotisation of ductus bursae. Probably the diverticulum pulsates during this stage, making the two combs of cornuti spread out; these actions were observed in males treated with DDVP. The anterior sclerotisation of ductus bursae may have evolved due to the movements of cornuti; their sharp tips would perforate an unsclerotised cuticle, and the females developed a sclerotisation as a protective measure. The function of the cornuti is less certain. Two possible functions (not incompatible) may be formulated: to push the sticky amorphous ejaculate further into the female genital tract, assisting the pumping function of the simplex; or the spreading of the cornuti may deform the anterior sclerotisation, stimulating the female. It should be emphasized that cornuti are incapable of individual movements, since only a few muscle fibres are attached to the bases of only the most proximal ones. The existence of some variation in the number of cornuti would be more compatible with a selective pressure of sexual nature. The idea that they represent a stimulus for the female would thus be reinforced. The thick exocuticle around the tormae of cornuti, forming a common plate around each row, makes them individually immovable. This way the intrinsic phallic muscle serves only to retract the vesica and its diverticulum, and the hypothetical stimulation of the female is achieved only through changing the hydrostatic pressure within the diverticulum. The second possibility would be related to a cryptic female choice mechanism [12]. In this case the female should be capable of sensing the deformations caused by the male, i.e., she should have receptors in the area. No specialised stretch receptors were observed, but even without such, proprioceptors (hypothetical, not observed) of the muscle fibers in the area may serve this function. In the second stage of copulation the vesica relaxes and the extended simplex is occupied by more viscous ejaculate (the collum of spermatophore), at that time functioning as a simple transport tube.

The experiments with eversion induced by DDVP and histology of everted vesicae clearly demonstrate the role of haemolymph pressure in the eversion process and contradicts the observations of Naumann [34] in *Zygaena*.

**Fig. 12:**
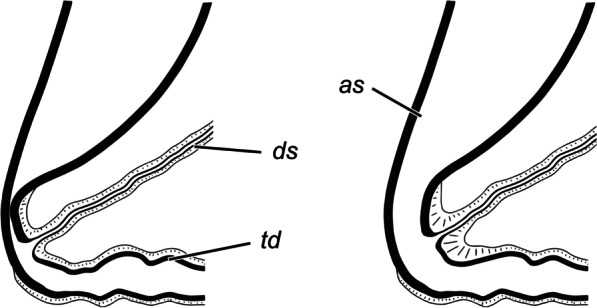
Hypothetical function of the anterior sclerotisation of ductus bursae as a valve (schematic). In closed position the muscularis (hatched) is relaxed, its contraction causes opening of the valve

The ductus bursae comprises distinct regions with certain functions. Here they are denoted with descriptive terms (apart of some easily recognisable parts), to avoid homologisation, which would be highly speculative given the enormous variation of these structures among Lepidoptera. The longitudinal region (posterior part, colliculum, longitudinal part and anterior sclerotisation) accommodates the phallus, and this is the site of direct interaction of the internal genitalia of both sexes. This part is probably also a subject of sexual selection: the vesica length is determined by the length of the longitudinal ductus bursae. The transverse region, a part of transporting ejaculated materials, accommodates the free portion of the collum, and similarly to the corpus bursae, its folds are smoothed when its volume increases. The transitional area between these regions is sharply bent at a right angle, and the ductus is strongly flattened at the angle and may serve as a valve (Fig. 12). It should be emphasised that the muscularis covers only the medial but not the lateral wall of the curvature. If the muscularis of this structure is relaxed, the valve is closed (i.e., the opposing sclerotised walls of the ductus are in tight contact). Contraction of the muscle fibres should cause the opening of the valve, considering their attachment. The possible function of the structure is to prevent the leakage of ejaculated materials back into the longitudinal part (which is a tube with considerable volume). The female may open the valve only during copulation, facilitating the male’s efforts. It can be speculated further that the action of the cornuti deforms the cuticle of the anterior sclerotisation, and that the female detects the deformation. In response, she opens the valve, allowing the transport of ejaculate. After complete insertion of the collum, the white material is transferred, and the muscularis of the valve relaxes and it closes. Eventually, the sperm is transported via the ductus seminalis; it is inserted in the same area, but anterior to the valve, supporting this putative function. A valve within the female genital tract has been recently described in *Leptophobia aripa* (Boisduval, 1836), though its position and hypothetical function are different [47]. This valve probably regulates the transfer of sperm from the spermatophore and is located anterior to the ductus seminalis, in contrast to the configuration in *T. viridana.* Anyway, both cases suggest that the ductus bursae is an active part of the process and not simply a genital passive duct.

The anatomy of the phallus and its female counterpart (i.e., longitudinal ductus bursae) in *T. viridana* are simple in comparison with some noctuids. In many of the latter the endophallus is a complex structure performing elaborate actions in two basically separate organs of the bursa copulatrix: the corpus bursae and the cervix bursae. Different parts of the vesica are everted in a specific sequence in the different parts of the female genital tract. The cornuti are involved in a complex manner of transportation of the collum, and in the precise placement of the frenum and the aperture of the spermatophore in relation to the position of the ductus seminalis insertion, which is not the case with *T. viridana* [4–6].

Given this complex scenario, the consideration of a mechanism based on the lock-and-key hypothesis seems an oversimplification ([32, 33] among others). Even if some spatial similarities may be detected in both male and female internal genitalia, these coincidences should not be necessarily interpreted as simply the result of an isolating process but the need of an efficient interaction.

### Formation of spermatophore

**Fig. 13:**
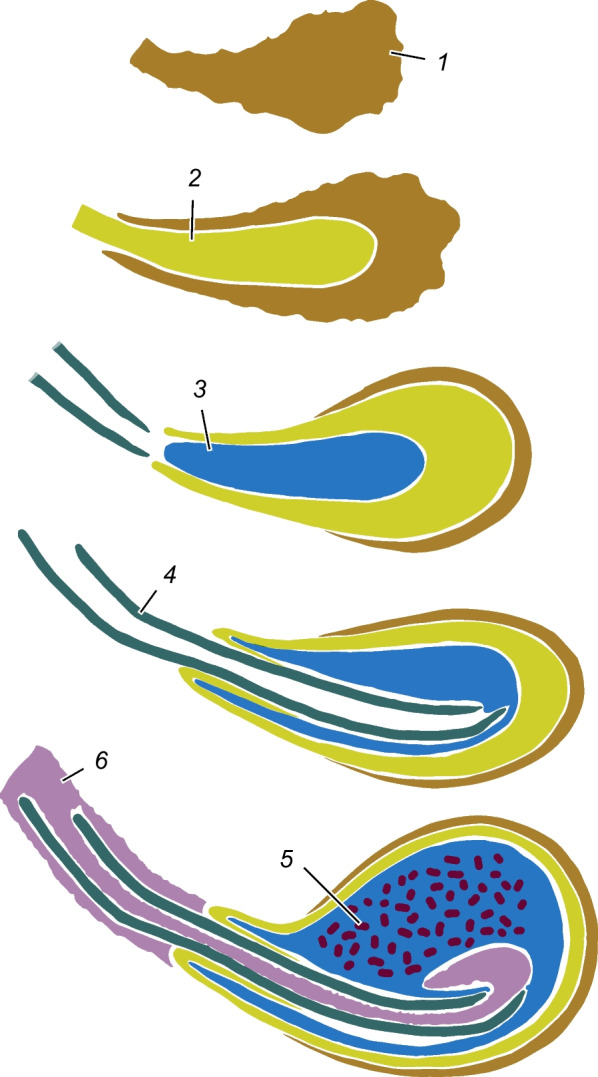
Formation of spermatophore (schematic). The numbers show the consequence of transferred materials. 1 brown material, 2 spermatophore corpus, 3 sperm sack, 4 collum, 5 sperm, 6 white material

(Fig. 13). The ejaculated materials either form compact structures or remain amorphous masses, similarly to other species in which the spermatophore has been studied. The most persisting elements of the lepidopterous spermatophore—the corpus, sperm sack and collum—were all found in *T. viridana* [27]. Notably, the aperture in this species is not specialised, and frenum is lacking. In *Manduca sexta* (Linnaeus, 1763), the transfer of a "yellow secretion" has been observed in the first stage of copulation; it is initially liquid but later turns to a cheese-like consistency. The entire spermatophore is embedded in this material [16], which is probably similar to either the white or the brown material in *T. viridana*. The main components of the spermatophore in *Bombyx mori* (Linnaeus, 1758) can be homologised with those in *T. viridana*. The pearly body, consisting of a compact lentiform mass and a thin envelop around the corpus, is probably homologous to the brown material. Part of the "wall" and the "outer matrix" appear to form the corpus, the "inner matrix" corresponds to the sperm sack, the "neck" is the collum, and the "soft plug" is the white material [36]. The white material in *T. viridana* occupies the entire ductus bursae and penetrates the collum and part of the sperm sack. It may serve at least two functions: providing nutrients for the female, and transportation of the sperm (like a soft plunger). It is absorbed in a mated female collected in the wild.

The consulted literature does not mention whether *T. viridana* is a monandrous or polyandrous species. In the museum specimens dissected by the authors we have never found more than one spermatophore. On the other hand, it is a dense, complex, and massive spermatophore that occupies the entire corpus bursae. It is questionable whether the female can accept more than one, preventing polyandry. Whether or not the male can produce a new spermatophore after copulation is even more unknown.

## Conclusions

This is the first attempt to give a complete functional interpretation of the genitalia in a Lepidoptera species. The combination of microscopy and X-ray techniques has revealed the best way to approach this challenge. Internal movements during copulation in *Tortrix viridana* are much more complex than the courtship. Sclerotised sacculi areas of the valvae are responsible for grasping the female abdomen meanwhile cuculli may provide sensorial information on female reactions. Ductus bursae includes distinct morphological as well as functional areas. The longitudinal part is the area of interaction with the male phallus. The transversal part is a rather simple duct ready to passively accommodate the collum. The transition between the two regions is a complex partially sclerotised area that plays the role of a valve. The spermatophore exhibits a complete structure except for the lack of frenum. It is formed by a strict sequence of deposits of materials from the outer layer to the internal cavity. Although our work contributes to clarify the mechanical interaction during copulation, future lines of research should address the study of the secretions involved, as well as the sensory interactions that certainly accompany these processes.

## Methods

### Gathering and fixation of samples

Approximately 100 pupae of *T. viridana* were collected in nature near Sofia (Bulgaria) on 22.v.2020. Each pupa was placed in a separate 15 ml Falcon tube along with a part of a rolled leaf. The tubes were inspected daily. After eclosion, moths were kept in two 3-L plastic boxes, one for females and the other for males. A piece of synthetic sponge (2 × 5 × 5 cm) and a cotton pad (ca. 2 cm^3^) soaked with 10% dextrose were placed on the bottom of the boxes to provide moisture and food. Copulation was facilitated in a third box in which one female and two males were placed. A short oak twig with several leaves was also put in the box to provide a substrate for the moths. The box was put in complete darkness in a closed cabinet at a temperature of 21 °C. Every two minutes the cabinet was open to check for a copulation. Each copulating pair was transferred carefully into a 15 ml Falcon tube and placed back into the cabinet. The first two pairs were left to complete the process, which was ca. 90 min under these conditions, and the moths were fixed eventually. One pair was fixed at every 10 ± 2 min after the onset of copulation, i.e., one couple at ca. 10, 20, 30, 40, 50, 60, 70, and 80 min. At the desired moment, 10 ml of freshly prepared and cooled (− 18 °C) Duboscq-Brasil fixative was poured on the copulating pair. The low temperature of the liquid caused rapid immobilization and death of the moths. After one minute, the pair along with the fixative, was transferred into a small glass dish, and the abdomens were gently injected with extra fixative. To avoid damage to the genitalia, a fine syringe (29G neddle) was inserted ventrally into the anterior part of the abdomen, and ca. 20–40 μl of fixative were injected. The samples were left for 24 h in the fixative, then the abdomens were cut off near the base and washed in 70% ethanol. The ethanol was changed twice a day until yellow colouring disappeared. Finally, they were stored in the same ethanol concentration for 4 months.

The non-copulated moths, as well as moths gathered in nature, were processed the same way as the copulating pairs.

### Micro-CT scanning

Prior to scanning, the samples needed to be contrasted. All procedures were performed at constant agitation on a rotator. As a first step, the samples were transferred into 5 ml tubes and washed with distilled water two times for 1 h, then stained in Lugol solution (0.66% (w/v) KI and 0.33% (w/v) I_2_) for 48 h [28]. The samples were washed three times for 1 h with distilled water, then immobilized in 2% hot agarose in 0.5 ml sterile centrifuge tubes. For scanning, an Xradia MicroXCT-400 (Carl Zeiss X-Ray Microscopy, Pleasanton, CA, USA) scanner was used. X-ray source settings were 60kVp/133µA, and projection images were acquired using the 4X detector assembly over a 360° rotation. Three of the copulating pairs (20, 50 and 70 min) were scanned with resolution of isotropic voxel size of 2.47 µm, they were used to produce high-resolution models, allowing reconstruction of finer structures (e.g., muscles). The other pairs (10, 30, 40, 60, 80 min) were scanned at lower resolution (4.95 µm isotropic voxel size) and used to study the changes in major structures during the process.

### Histology

After scanning, the samples were processed to obtain histological sections. The agarose was peeled carefully from the abdomens in copula with forceps and a needle. The iodine staining was washed out with 70% ethanol for 14 days, with daily change of the solvent. Then the samples were dehydrated with ethanol series, cleared with xylene, and embedded in Paraplast Plus®. Six samples were sectioned parallel to the sagittal plane, and two were sectioned transversely, all at 5 μm thickness. A spermatophore of a female fixed immediately after copulation and an everted vesica of a male were dehydrated with acetone and embedded in methacrylates mixture (74% butyl methacrylate, 24% methyl methacrylate, 1% benzoyl peroxide, 1% dicyclohexyl phthalate), then sectioned with a tungsten carbide knife at 2 μm. The embedding media from all sections was dissolved, and they were stained with Lower's trichrome [26]. The slides were eventually mounted in Euparal.

### Cuticular preparations

Specimens gathered in nature and fixed in Bouin's fixative or dry set specimens were macerated in hot 10% KOH solution until only cuticle remained, then the genitalia were dissected. Only the females were stained with 0.03% chlorazol black E for 30–60 s. All samples were washed two times with 5% ethanol, dehydrated in 70% and 100% (two times) ethanol, 1 min in each, and eventually transferred in Euparal essence. Two males and two females were processed this way. A small glass Petri dish (⌀ = 15 mm, h = 6 mm) glued with Euparal to a standard slide and filled with Euparal essence was used for photography of the cuticular preparations without compression or deformation, freely floating in the liquid. The dish was photographed under a compound microscope.

### Three-dimensional reconstruction

The scans were processed with Aviso software in the FUNEVOL lab in Paris (UMR7179) and three-dimensional models were obtained. The scans were compared with corresponding histological sections to enhance the recognition of the observed tissues and structures. The muscles and sclerotised skeletal parts were segmented mainly with the automatic functions of the software. The low contrast and very small thickness of the membranous structures demanded extensive manual segmentation.

### Muscle staining and CLSM

The visualization of the muscles via confocal laser scanning microscopy (CLSM) follows Zlatkov et al. [50] with some modifications. Initially, the samples were fixed in Bouin’s fluid and after washing, stored in ethanol. Re-fixation was conducted in 4% paraformaldehyde in 0.2 M phosphate buffer with 0.3% Triton X-100 (PBST) at room temperature for one hour. Then the samples were stained following Siwanowicz and Burrows [43] with Texas Red™-X Phalloidin (1:50, Thermo Fisher Scientific, #00033) and Calcofluor White (0.1 mg/ml, Sigma-Aldrich, #18909) at 4 °C with agitation for 4–5 days. After that, samples were subjected to the dehydration process and cleared overnight in methyl salicylate 98% and eventually transferred to Euparal permanently, to visualize under confocal laser scanning microscope (Olympus FV1000) at 4X, 10X and 20X. The CLSM images were obtained and stacked with Olympus-FluoView (FV10-ASW) software. The raw images were processed using image analysis software Fiji-ImageJ [38].

### Scanning electron microscopy

The same female genital tracts that had been used for confocal microscopy were used to visualize muscles using a scanning electron microscope (SEM). They were manipulated under stereomicroscope and gently washed with ethanol and water to remove all remains of previous chemicals that might interfere. The pieces were then fixed by immersion in Karnovsky’s fixative [21] containing Triton X-100 0.1% for 24 h. Fragments for SEM preparation were washed in distilled water and progressively dehydrated through a graded ethanol series. Pieces were placed inside microporous specimen capsules (30 μm pore size) immersed in absolute ethanol. Critical point drying was performed in a Leica EM CPD300. The obtained fragments were arranged on SEM aluminium stubs using carbon tape and coated with Au/Pd sputtered in argon gas. Observation and photography were performed in a SEM model SCIOS 2 (Thermo Fisher).

### Treatment with 2,2-dichlorovinyl dimethyl phosphate (DDVP)

This compound was applied to virgin males to induce eversion of the vesica [9], and the dosage followed Zlatkov [49]. Eleven male moths were exposed to the chemical for 2 min, then were immobilised with the ventral side up on a small piece of polyethylene foam with 0.25 mm minuten pins driven through the wings. The ejaculated material was removed immediately with a forceps; if not removed, it hardens and prevents eversion. The males were fixed 60 min after onset of eversion in the same manner as the copulating pairs.

### Observation and photomicrography

The eversion in vivo, fixations, and dissections were observed and documented under a Stemi 2000-c stereo microscope with a DSLR camera (Canon EOS 1300D) attached. A compound microscope Amplival (Carl Zeiss Jena) equipped with a DSLR camera (Canon EOS 2000D) and polarising device was used for examination of some samples and photography.

### Final image processing

All images were edited with Photoshop (Adobe). The videos were edited with Movie Maker (Microsoft).

### Abbreviations and terminology

The nomenclature of the skeleton follows mainly Klots [22], and for certain structures descriptive terms have been created. The musculature nomenclature follows Kuznetsov and Stekolnikov [24], but some muscles are homologised after Kristensen [23]. The term “internal genitalia” is used to refer to the phallus, ductus bursae and bursa copulatrix, and “external genitalia” to all other cuticular elements directly related to the reproduction.

## Supplementary Information

**Additional file 1.** Male genitalia musculature of *Tortrix viridana* L.

**Additional file 2.** Female genitalia musculature of *Tortrix viridana* L.

**Additional file 3.** Position of the genitalia of *Tortrix viridana* L. during copulation (only skeletal structures).

**Additional file 4.** Movements of the vesica of *Tortrix viridana* L.

## Data Availability

The image data used and analysed during the current study are available from the corresponding author on reasonable request. The samples are preserved in the Institute of Biodiversity and Ecosystem Research, Sofia, Bulgaria, and Institut Cavanilles de Biodiversitat i Biologia Evolutiva, Universitat de València, Paterna, Spain.

## Abbreviations

*aa*: Apophysis anterior
*ac*: Anal cone
*an*: Antrum
*ap*: Apophysis posterior
*as*: Anterior sclerotisation of ductus bursae
*br*: Brachiola
*cb*: Corpus bursae
*cl*: Colliculum
*co*: Cornuti
*cp*: Coecum penis
*cs*: Costa
*cu*: Cucullus
*de*: Ductus ejaculatorius
*di*: Diverticulum of vesica
*dl*: Dorsal lobe of papilla analis
*ds*: Ductus seminalis
*gp*: Gonopore
*I*: Insertion of muscle
*ju*: Juxta
*ld*: Longitudinal part of ductus bursae
*maa. 7–8*: Musculus apophysalis anterior 7–8
*maa. 7–9*: Musculus apophysalis anterior 7–9
*map. 7–8a*: Musculus apophysalis posterior 7–8 anterior
*map. 7–8p*: Musculus apophysalis posterior 7–8 posterior
*map. 8–9*: Musculus apophysalis posterior 8–9
*ma-s. 8–9*: Musculus apophyso-sternalis
*mg. ed-l*: Musculus gonopodialis externus dorsolateralis (= m_2_)
*mi. 8–9*: Musculus interapophysalis 8–9
*ml*: Median lobe of papilla analis
*mlm. a*: Musculus laminae mediale anterior (= m_3_)
*mom.*: Muscle of median oviduct
*mph. ea*: Musculus phallicus externus anterior (= m_6_)
*mph. ep*: Musculus phallicus externus posterior (= m_5_)
*mph. il-t*: Musculus phallicus internus longitudinalis (= m_21_)
*ms. 7–8a*: Musculus sternalis intersegmentalis longitudinalis 7–8 anterior
*ms. 7–8p*: Musculus sternalis intersegmentalis longitudinalis 7–8 posterior
*ms. 8–9*: Musculus sternalis intersegmentalis longitudinalis 8–9
*ms-t. 8–9*: Musculus sternotergalis intersegmentalis 8–9
*mt. 10–11*: Musculus tergalis intersegmentalis 10–11 (= m_10_)
*mt. 7–8*: Musculus tergalis intersegmentalis 7–8
*mt. 9–10*: Musculus tergalis intersegmentalis 9–10 (= m_1_)
*mtd-v. 8*: Musculus tergalis intrasegmentalis dorsoventralis 8
*mt-s. 8–9*: Musculus tergosternalis intersegmentalis 8–9
*O*: Origin of muscle
*os*: Ostium ductus bursae
*pb*: Processus basalis valvae
*pd*: Posterior part of ductus bursae
*ph*: Phallus
*S7, S8*: Sternite 7, 8
*sa*: Sacculus
*sf*: Sclerotised fold of papilla analis
*si*: Signum
*so*: Socius
*sp*: Spermatheca
*st*: Sterigma
*T7, T8*: Tergite 7, 8
*td*: Transversal part of ductus bursae
*te*: Tegumen
*ve*: Vesica
*vi*: Vinculum
*vl*: Ventral lobe of papilla analis
